# Real-World safety, patient-reported outcomes, and glycemic metrics with a 7-Day extended infusion set in adults with Type 1 diabetes using automated insulin delivery systems

**DOI:** 10.1007/s12020-026-04743-z

**Published:** 2026-08-21

**Authors:** María de la O Nieto de la Marca, Pablo Fernández Velasco, Sofía Del Amo Simón, Marta Sánchez Ibáñez, Ana Collantes Matallana, Emilia Gómez Hoyos, Beatriz Torres Torres, Ana Ortolá Buigues, Daniel Antonio de Luis Román, Gonzalo Díaz Soto

**Affiliations:** 1 Centro de Investigación de Endocrinología y Nutrición. Faculty of Medicine, University of Valladolid, Valladolid. Spain, Valladolid, Spain; 2 Department of Endocrinology and Nutrition, Hospital Clínico Universitario de Valladolid, Valladolid, Spain; 3 Valladolid Health Research Institute (IBioVALL), C. Rondilla Sta. Teresa, 47010 Valladolid, Spain; 4 Centro de Investigación Biomedica en Red (CIBEROBN) de la Obesidad y Nutrición. Instituto de Salud Carlos III, Madrid, Spain

**Keywords:** Type 1 diabetes, MiniMed 780G, Extended infusion set, Hybrid closed loop, Treatment satisfaction

## Abstract

**Background:**

Extended-wear infusion sets (E-iS) allowing up to 7 days of catheter use have recently become available. Real-world evidence of contemporary automated insulin delivery (AID) systems using E-iS remains limited.

**Method:**

A prospective non-randomized single-arm real-world study in adults with type 1 diabetes using the MiniMed-780G AID system who transitioned from conventional 3-day infusion sets to E-iS was conducted. Clinical variables, device settings, adverse events, and patient-reported outcomes were assessed at baseline, 3 and 6 months.

**Results:**

65 participants were included, 58 completed 6 months of follow-up.Mean catheter wear time increased from 3.6 ± 1.1 to 6.1 ± 2.3 days,(p < 0.001), and reservoir change interval increased from 3.5 ± 1.0 to 5.2 ± 2.3 days,(p < 0.001). Glycemic control remained overall stable, although small changes were observed in some CGM metrics (Time in range-TIR: 80.0 ± 7.1% to 78.0 ± 10.0%, p = 0.014) and slight increases in time above range, while hypoglycemia exposure remained unchanged. HbA1c decreased from 6.8 ± 0.5% to 6.4 ± 1.0%, (p = 0.03). Total daily insulin dose did not change, but insulin use related to infusion set management decreased significantly. Pain at cannula insertion improved from 2.1 ± 1.5 to 1.2 ± 1.1,(p < 0.001), and treatment satisfaction increased from 8.2 ± 1.9 to 9.0 ± 1.2,(p = 0.012). No episodes of severe hypoglycemia or diabetic ketoacidosis occurred. Cannula wear duration was strongly correlated with reservoir change interval (r = 0.712, p < 0.001). At 6 months, 92.3% of participants continued using the E-iS, with 48.3% maintaining the full 7-day wear duration.

**Conclusions:**

In real-world use with an AID system, the E-iS significantly prolonged catheter wear duration and reservoir change intervals while remaining safe and well tolerated. It was associated with high satisfaction and stable glycemic outcomes, supporting E-iS as a practical strategy to reduce the burden of infusion set changes in routine clinical practice.

## Introduction

Type 1 diabetes mellitus (T1DM) is a chronic autoimmune disease characterized by hyperglycemia resulting from the destruction of pancreatic beta cells [1]. Lifelong physiological insulin replacement is required, with the primary goal of optimizing glycemic control. From the time of diagnosis, this approach is essential to reduce the risk of both acute events and long-term microvascular and macrovascular complications [2].

Insulin delivery and glucose monitoring technologies have evolved markedly over the past decade. Automated insulin delivery (AID) systems integrate algorithms with continuous glucose monitoring (CGM) to regulate insulin infusion. They are currently considered the standard of care for individuals with T1DM who have the capacity and willingness to use them [3]. AID systems have been shown to improve metabolic control while reducing the burden of daily self-management [4,5].

Although AID automation continues to increase, all commercially available systems require user input for meal announcement and physical activity. In addition, structured therapeutic education and ongoing training remain essential to ensure safe and effective device use [6]. Particular attention must be paid to infusion set management, including appropriate site rotation and timely replacement. Infusion-set-related problems and inadequate replacement can lead to acute complications that are not usually observed with multiple daily injections [7]. Moreover, different infusion set types are available, with cannulas that vary in material and length, and selection should be individualized according to each person’s characteristics and needs [8].

The MiniMed 780G AID system (Medtronic MiniMed, Northridge, CA, USA) is a hybrid closed-loop system that uses a predictive algorithm with adaptive proportional integral derivative control to automatically adjust basal insulin delivery and provide correction boluses based on CGM data. Its effectiveness has been demonstrated in clinical trials and real-world studies [9]. However, complications related to catheter use and replacement have received comparatively less attention with this and other AID systems. Since reporting of catheter-related events is not automated in download platforms, they are often underreported.

Catheter wear in AID therapy is associated with a range of potential complications. The prevalence of insulin related lipohypertrophy may exceed 40% and is directly related to treatment duration [10]. Catheter wear beyond day three has been associated with a higher risk of diabetic ketoacidosis, unexplained hyperglycemia, severe hypoglycemia, and other adverse events related to infusion set and catheter use [11]. Reported infusion set adverse events include catheter knots, kinking on insertion or during wear, and accidental traction. Cannula-related and local infusion-site events may include insulin leakage, bleeding, erythema, induration, infection, pain, and unexplained hyperglycemia. Adhesive-related problems may also occur, including patch loosening with heat exposure or prolonged wear, which may lead to local pruritus, erythema, and pain; however, these symptoms may also reflect local infusion-site or cannula-related reactions. Insulin leakage may occur after catheter disconnection, accidental cannula traction, or under the cannula patch, while bleeding within the cannula or at the insertion site may cause pain or obstruct insulin flow [12].

In some individuals, infusion set, cannula, and insertion site adverse events can lead to discontinuation of AID therapy. A median discontinuation rate of approximately 7% due to infusion set, cannula, and insertion site complications has been reported [13].

In 2021, the US Food and Drug Administration (FDA) approved the Medtronic Extended infusion set (E-iS), which is also CE-marked, allowing catheter wear for up to 7 days, twice the duration of conventional 3-day infusion sets. It also theoretically allows synchronization of infusion set replacement with reservoir replacement, provided weekly insulin requirements are <300 IU/week. Previous non-randomized studies have associated the MiniMed Extended system in non-AID pump therapy, including the 670 G system, with greater convenience and improved quality of life without deterioration in glycemic control [14]. However, robust real-world evidence evaluating the safety, effectiveness, durability, glycemic outcomes, discontinuation rates, infusion-site complications, and patient-reported outcomes associated with extended-wear infusion sets in contemporary AID systems remains limited.

The present study evaluates the safety and effectiveness of the Medtronic Extended infusion set with a wear time of up to 7 days in AID systems in real-world practice. It also assesses treatment satisfaction and quality of life after transition from conventional 3-day infusion sets (MiniMed Mio Advance and MiniMed Quick Set).

## Materials and methods

A prospective non-randomized single-arm, real-world observational study was conducted in routine clinical practice with participants serving as their own control. We included all adult patients followed at a tertiary care hospital with a diagnosis of T1DM who were users of the Medtronic MiniMed 780G AID system together with the Guardian 4 CGM sensor. Eligible participants had at least 12 months of experience using the AID system in automatic mode and were using a straight infusion catheter, either 6 mm or 9 mm, with a wear duration of up to three days. All patients signed an informed consent form before entering the study. Refusal to provide consent was an exclusion criterion. The protocol was approved by the Institutional Clinical Research Ethics Committee (PI 25/49 C), and the study was conducted in accordance with the Declaration of Helsinki.

**Fig. 1:**
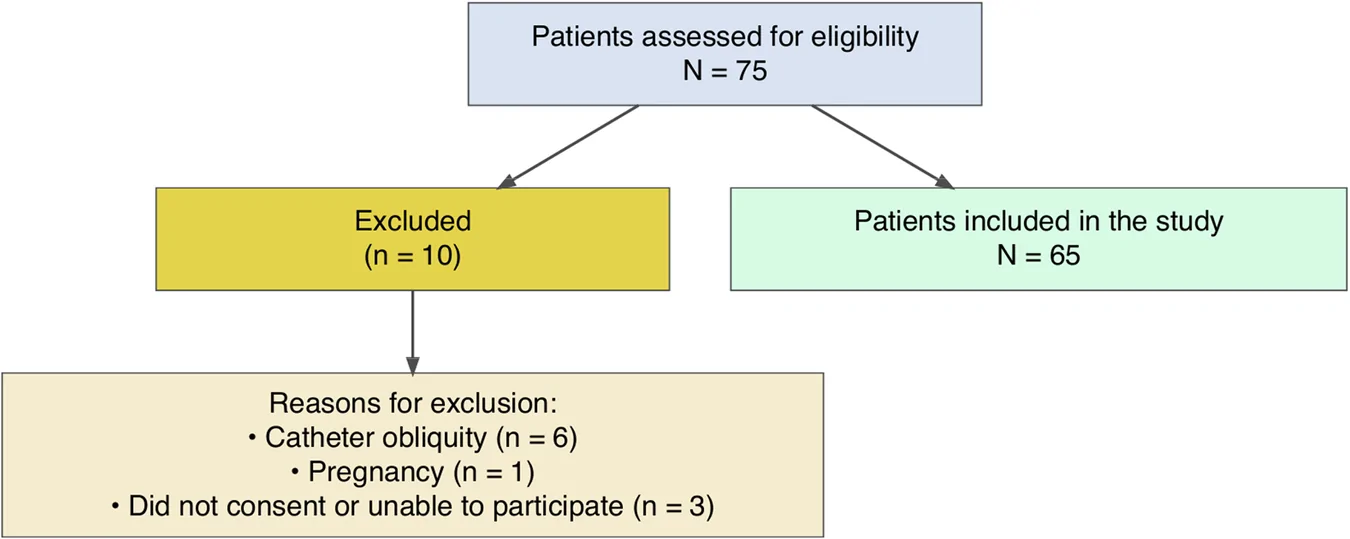
Patient flow diagram. Flow diagram of patient eligibility, exclusion, and inclusion in the study

Exclusion criteria were use of an angled catheter, severe hypoglycemia within the previous six months, or severe hyperglycemia with diabetic ketoacidosis requiring hospital admission within the previous two years. Additional exclusion criteria were pregnancy or pregnancy planning, insufficient AID use, or less than 85% time in automatic mode. A flow diagram summarizing participant selection and inclusion and exclusion criteria is presented in Fig. 1.

### Educational intervention and follow up

A structured educational intervention and transition to the E-iS were performed according to the center´s internal protocol during March and April 2025 through an initial group visit. All participants initiated the new infusion set during this session. Step-by-step training addressed infusion set replacement and management of acute complications, including hypoglycemia, hyperglycemia, and ketosis. Participants were instructed that reservoir replacement could be performed independently of infusion set replacement when insulin was depleted before completion of the intended 7-day wear period, with the aim of supporting completion of the full intended catheter wear duration independently of reservoir changes. Participants were also instructed to prospectively record in detail any catheter or infusion set-related complication using an application designed for this purpose.

Follow-up included a telephone contact at one month for all participants using the device download platforms. Two individual in-person visits were conducted at three and six months after initiation of the new infusion set. At the 6-month visit, continuation or discontinuation of the E-iS was formally assessed.

### Data collection and outcomes

Sociodemographic variables were recorded at baseline. Anthropometric, clinical, metabolic, laboratory, and CGM-derived variables were collected at baseline and at three and six months. Data on device configuration, infusion set characteristics, adverse events, and satisfaction were also collected at the same time points.

HbA1c was measured using a turbidimetric inhibition immunoassay standardized to the National Glycohemoglobin Standardization Program (NGSP), (Roche Diagnostics, Geneva, Switzerland). Glycemic metrics were obtained from the AID system data download platform for all patients attending each follow up visit. The report included the 14 days preceding each visit. Glycemic metrics included mean glucose (mg/dL), glucose management indicator (GMI, %), coefficient of variation (CV, %), and percentages of time in range (TIR), time in tight range (TiTR), time above range (TAR), and time below range (TBR). TBR was classified as very low for glucose <54 mg/dL and low for 54–70 mg/dL. TIR was defined as 70–180 mg/dL. TAR was classified as high for 181–250 mg/dL and very high for >250 mg/dL.

Device use variables included time in automatic mode, weekly time in manual mode, total daily insulin dose (TDD), daily bolus insulin dose, daily basal insulin dose, number of days using the same catheter, number of days using the same reservoir, and device configuration variables including glucose target and active insulin time.

Insulin utilization data were obtained from the centralized electronic prescription system of the National Health Service. The number of days covered by 1500 IU, corresponding to the available dispensing unit for rapid-acting insulin in Spain, was calculated at baseline and during follow-up as an indirect estimate of insulin consumption related to infusion set changes, including priming and reservoir replacement procedures.

Infusion set related variables included baseline infusion set and cannula type, pain at insertion and pain during wear, satisfaction with the new infusion set, and frequency of adverse events with the new infusion set. Catheter- or infusion set-related adverse events were prospectively recorded by participants using the study-specific application whenever they occurred and were reviewed at each follow-up visit. Patient-reported outcomes and satisfaction questionnaires were completed at the scheduled study visits. Adverse events included occlusion alarms, accidental removal, lipohypertrophy, skin marks, skin wounds, pruritus, local inflammatory/infectious reaction, and unexplained hyperglycemia. Unexplained hyperglycemia was defined as sustained hyperglycemia not attributable to meals, missed boluses, intercurrent illness, or other identifiable causes, and considered by the participant or clinical team to be potentially related to infusion set performance. Adverse events were expressed as the percentage of patients experiencing each event during follow-up.

### Patient reported outcomes

Quality of life was assessed using the validated Spanish version of the Diabetes Quality of Life (EsDQoL) questionnaire [15], which analyzes satisfaction, impact, social concern, and diabetes-related concern, in which lower scores indicate better quality of life (range: 43–215). The Clarke questionnaire was also used to assess hypoglycemia awareness, with scores of 1–2 indicating normal awareness, 3 indicating indeterminate awareness, and >3 indicating impaired awareness [16]. Additionally, a visual analog scale (VAS) was used to evaluate pain and satisfaction (range: 0–10), with 10 indicating maximum pain intensity and the highest level of satisfaction, respectively.

### Statistical analysis

The sample size was estimated for the endpoint, change in catheter wear time from baseline to follow-up. Assuming a clinically relevant mean increase of 2.0 days, a conservative standard deviation of within-subject paired differences of 3.0 days, a two-sided α of 0.05, and 90% power, a minimum of 24 participants was required. Allowing for a 15% attrition rate, at least 29 participants were needed.

Quantitative variables are presented as mean (SD) when normally distributed or as median with interquartile range when not normally distributed. Qualitative variables are presented as percentages.

Normality of quantitative variables was assessed using the one-sample Kolmogorov–Smirnov test. Comparisons between two independent groups were performed using a two-sided Student’s t test for normally distributed variables or the Mann–Whitney U test for non-normally distributed variables. Categorical variables were compared using the χ² test or Fisher’s exact test when appropriate for comparisons between independent groups. Associations between normally distributed continuous variables were assessed using the Pearson correlation coefficient.

Changes across study time points (baseline, 3 months, and 6 months) were evaluated using repeated-measures analysis of variance (ANOVA), with Bonferroni-adjusted post hoc pairwise comparisons when the global test was significant. For paired categorical comparisons across time points, McNemar’s test was used. All analyses were performed according to the intention-to-treat principle. Missing data were handled using the last observation carried forward method.

A p value < 0.05 was considered statistically significant. Analyses were conducted using SPSS version 25.0 (IBM Corp., Armonk, NY, USA) and RStudio (Posit Software, PBC, Boston, MA, USA).

## Results

A total of 65 individuals with T1DM were included, of whom 45 (69.2%) were women. Mean age was 39.8 (11.8) years, and baseline BMI was 27.4 (7.1) kg/m^2^. Mean HbA1c was 6.8 (0.5) %, and time since T1DM diagnosis was 25.0 (11.0) years. Participants had substantial prior experience with technology, with a mean of 114 (54.0) months of CSII use and 33 (20.0) months of AID use. Baseline demographic and clinical characteristics, including education level, employment status, microvascular complications, previous infusion set type, cannula length, and insulin type, are summarized in Table 1.

**Table 1:** Baseline Characteristics of the Study Population

Baseline characteristics	Mean ± SD or n (%)
**Number of participants**	65
**Women**	45 (69.2%)
**Age, years**	39.8 ± 11.8
**Weight, kg**	75.5 ± 15.4
**BMI, kg/m²**	27.4 ± 7.1
**Time since T1DM diagnosis, years**	25 ± 11
**Time since CSII initiation, months**	114 ± 54
**Time since CGM initiation, months**	33 ± 20
**HbA1c, %**	6.8 ± 0.5
**Education level**	
Primary education	4 (6.2%)
Secondary education	6 (9.2%)
High school or higher vocational training	24 (36.9%)
University education	31 (47.7%)
**Microvascular complications**	
Retinopathy	12 (18.5%)
Kidney disease	3 (4.6%)
Microalbuminuria	2 (3.1%)
**Employment status**	
Not working	17 (26.2%)
Fixed shifts	36 (55.4%)
Rotating shifts	12 (18.5%)
**Infusion set used**	
MiniMed Quick-set	54 (83.1%)
MiniMed Mio Advance	11 (16.9%)
**Cannula length**	
6 mm	34 (52.3%)
9 mm	31 (47.7%)
**Insulin type used in CSII**	
Insulin aspart	3 (4.6%)
Insulin lispro	28 (43.1%)
Faster insulin aspart	34 (52.3%)

Data are presented as mean ± SD or n (%)

*BMI*, body mass index; *T1DM*, type 1 diabetes mellitus; *CSII*, continuous subcutaneous insulin infusion; *CGM*, continuous glucose monitoring

At baseline, participants showed high AID use and good glycemic control, with 94.8 (3.2)% time in automatic mode, mean glucose of 141.7 (10.6) mg/dL, TIR of 80.0 (7.1)%, and low hypoglycemia exposure (TBR < 70 mg/dL of 2.0 (1.4) % and TBR < 54 mg/dL of 0.6 (0.6) %). No participant reported severe hypoglycemia within the previous 6 months or DKA requiring hospital admission within the previous 2 years. Baseline device settings, glycemic metrics, insulin doses, infusion set variables, and adverse events are shown in Table 2, while patient-reported outcomes are summarized in Table 3.

**Table 2:** Glycemic and Device Related Outcomes at Baseline and Follow Up

Parameter	Baseline (n = 65)	3 months (n = 61)	6 months (n = 58)	P value
**HbA1c, %**	6.8 ± 0.5	6.9 ± 1.0	6.4 ± 1.0	0.03 (*)
**Weight, kg**	75.5 ± 15.4	74.5 ± 15.4	74.1 ± 15.4	ns
**BMI, kg/m2**	27.4 ± 7.1	26.8 ± 5.2	27.4 ± 9.4	ns
**AID glucose target**				
**100 mg/dL**	46 (70.8%)	46 (75.4%)	42 (72.4%)	
**110 mg/dL**	11 (16.9%)	10 (16.4%)	11 (19%)	
**120 mg/dL**	8 (12.3%)	5 (8.2%)	5 (8.6%)	
**Active insulin time**				
**2.0 h**	39 (60%)	36 (59%)	35 (60.3%)	
**2.3 h**	2 (3.1%)	3 (4.9%)	3 (5.2%)	
**2.5 h**	16 (24.6%)	14 (22.9%)	11 (19%)	
**2.8 h**	2 (3.1%)	2 (3.3%)	2 (3.4%)	
**3.0 h**	5 (7.7%)	6 (9.9%)	7 (12.1%)	
**4.0 h**	1 (1.5%)	0 (0%)	0 (0%)	
**Mean glucose, mg/dL**	141.7 ± 10.6	142.8 ± 14.8	145.3 ± 14.2	0.024 (*)
**GMI, %**	6.6 ± 0.9	6.7 ± 0.3	6.8 ± 0.3	ns
**CV, %**	30.0 ± 4.3	29.9 ± 5.8	30.9 ± 4.8	ns
**TIR, %**	80.0 ± 7.1	80.7 ± 8.7	78.0 ± 10.0	0.014 (*)
**TBR** < **70 mg/dL, %**	2.0 ± 1.4	1.4 ± 1.3	1.2 ± 1.4	ns
**TBR** < **54 mg/dL, %**	0.6 ± 0.6	0.15 ± 0.4	0.3 ± 0.7	ns
**TAR** > **180 mg/dL, %**	15.0 ± 6.2	15.0 ± 6.7	17.0 ± 6.9	0.03 (*)
**TAR** > **250 mg/dL, %**	2.4 ± 2.1	3.0 ± 2.7	3.5 ± 4.1	0.01 (*)
**TiTR, %**	54.0 ± 10.3	55.0 ± 12.0	52.0 ± 11.8	0.02 (*)
**Time in automatic mode, %**	94.8 ± 3.2	92.5 ± 12.7	94.4 ± 4.1	ns
**Total daily insulin dose, IU**	45.0 ± 19.4	47.2 ± 21.3	45.8 ± 22.8	ns
**Daily bolus insulin dose, IU**	27.0 ± 13.7	26.2 ± 13.2	27.6 ± 14.5	ns
**Daily basal insulin dose, IU**	18.0 ± 5.7	21.0 ± 8.1	18.2 ± 8.8	ns
**Catheter change interval, days**	3.6 ± 1.1	6.1 ± 2.0	6.1 ± 2.3	<0.001 (#,*)
**Reservoir change interval, days**	3.5 ± 1.0	5.3 ± 2.0	5.2 ± 2.3	<0.001(#,*)
**Adverse event, %**				
Occlusion alarms	15.4%	18.0%	8.6%	ns
Accidental removal	40.0%	44.3%	39.7%	ns
Lipohypertrophy	23.1%	16.4%	25.9%	ns
Skin marks	41.5%	44.3%	39.7%	ns
Skin wounds	16.9%	16.4%	17.2%	ns
Pruritus	44.6%	39.3%	34.5%	ns
Local inflammatory/infectious reaction	13.8%	11.5%	5.2%	ns
Unexplained hyperglycemia	23.1%	31.1%	25.9%	ns

Data are presented as mean ± SD or n (%)

P values for continuous variables correspond to repeated-measures ANOVA analysis. Significant Bonferroni-adjusted pairwise comparisons are indicated as follows: # baseline vs 3 months; * baseline vs 6 months. ns, not significant. Adverse events are expressed as the percentage of patients experiencing each event during each follow-up period

*AID*, automated insulin delivery; *BMI*, body mass index; *CV*, coefficient of variation; *GMI*, glucose management indicator; *TAR*, time above range; *TBR*, time below range; *TiTR*, time in tight range; *TIR*, time in range

**Table 3:** Patient Reported Outcomes at Baseline and Follow Up

Questionnaire or domain	Baseline (n = 65)	3 months (n = 61)	6 months (n = 58)	P value
**VAS (0 to 10)**				
**Pain during cannula insertion**	2.1 ± 1.5	1.1 ± 1.0	1.2 ± 1.1	<0.001 (#,*)
**Pain during infusion set wear**	1.2 ± 1.1	0.9 ± 1.1	0.96 ± 1.0	ns
**Satisfaction with current infusion set**	8.2 ± 1.9	9.0 ± 1.6	9.0 ± 1.2	0.012 (#,*)
**EsDQoL (range 43 to 215)**				
**Satisfaction**	30.4 ± 12.2	27.9 ± 7.9	27.9 ± 7.6	0.03 (#,*)
**Impact**	33.3 ± 14.7	31.1 ± 8.1	32.9 ± 9.6	ns
**Social concern**	11.0 ± 3.9	11.5 ± 3.6	11.1 ± 3.6	ns
**Diabetes related concern**	8.2 ± 2.8	7.9 ± 2.4	8.0 ± 2.3	ns
**Total**	82.9 ± 33.6	78.4 ± 22.0	79.9 ± 23.1	ns
**Clarke categories**				
**Negative**	44 (67.7%)	41 (67.2%)	44 (75.9%)	ns
**Positive**	11 (16.9%)	9 (14.7%)	9 (15.5%)	
**Indeterminate**	10 (15.4%)	11 (18.1%)	5 (8.6%)	
**Clarke score, R**	1.8 ± 1.7	1.58 ± 1.57	1.4 ± 1.6	ns

Data are presented as mean ± SD or n (%)

P values correspond to repeated-measures ANOVA analysis. Significant Bonferroni-adjusted pairwise comparisons are indicated as follows: # baseline vs 3 months; * baseline vs 6 months. ns, not significant

EsDQoL, Spanish version of the Diabetes Quality of Life measure. VAS, visual analog scale

Overall, 58 participants completed follow-up and 7 discontinued. Discontinuations were due to adverse events before month 6 in 4 participants, all of whom returned to their previous infusion set, and loss to follow-up in 3 participants.

After switching to the E-iS, catheter wear time increased significantly over follow-up. Mean wear time increased from 3.6 (1.1) days at baseline to 6.1 (2.3) days at 6 months (p < 0.001). Similarly, the reservoir change interval increased from 3.5 (1.0) to 5.2 (2.3) days (p < 0.001). Bonferroni-adjusted comparisons confirmed significant increases from baseline to both 3 and 6 months.

HbA1c changed significantly over time, decreasing from 6.8 (0.5) % at baseline to 6.4 (1.0)% at 6 months (p = 0.03). Glycemic outcomes remained overall stable, although several CGM metrics showed modest but statistically significant changes over time. Mean glucose increased from 141.7 (10.6) to 145.3 (14.2) mg/dL (p = 0.024). TIR decreased from 80.0 (7.1)% to 78.0 (10.0)% (p = 0.014). TAR > 180 mg/dL increased from 15.0 (6.2)% to 17.0 (6.9)% (p = 0.03), and TAR > 250 mg/dL increased from 2.4 (2.1)% to 3.5 (4.1)% (p = 0.01). TiTR decreased from 54.0 (10.3)% to 52.0 (11.8)% (p = 0.02). CV and TBR remained stable over the study period.

Device settings remained unchanged throughout follow-up, including glucose target and active insulin time. Total daily, basal, and bolus insulin doses also remained stable. Based on prescription program estimates, insulin use related to infusion set changes decreased due to fewer priming and reservoir emptying procedures. Specifically, 1500 IU covered 18.4 (9.4) days at baseline compared with 26.1 (10.7) days after switching to the E-iS (p < 0.001).

At 6 months, pain at cannula insertion improved from 2.1 (1.5) to 1.2 (1.1) (p < 0.001). Satisfaction increased from 8.2 (1.9) to 9.0 (1.2) (p = 0.012) and the EsDQoL satisfaction subscale improved from 30.4 (12.2) to 27.9 (7.6) (p = 0.03). Other patient-reported outcomes remained stable, with no deterioration in the evaluated domains.

At 6 months, 92.3% of participants continued using the Extended infusion set. Five participants (7.7%) returned to the previous infusion set, and two additional participants withdrew from study follow-up while continuing to use the E-iS. Reasons for returning to the previous infusion set included adhesive failure with insulin leakage in 2 cases, persistent nodules and induration at the insertion site in 1 case, hyperglycemia beginning on day 2 in 1 case, and wear duration <5 days in 1 case. No participant experienced severe hypoglycemia or DKA requiring hospital admission after switching during the study period.

Adverse events are summarized in Table 2. No significant changes were observed over time in any adverse-event category. Occlusion alarms were reported by 15.4%, 18.0%, and 8.6% of participants at baseline, 3 months, and 6 months, respectively. Unexplained hyperglycemia was reported by 23.1%, 31.1%, and 25.9%, respectively. Skin-related and local inflammatory/infectious events remained stable during follow-up.

Among participants assessed at 6 months, 65.6% maintained the infusion set for ≥5 days. Compared with those using the catheter for <5 days, no significant differences were observed except for insulin dose. Total daily insulin dose was lower in the ≥5 days group at 38.9 (18.5) vs 58.8 (26.8) IU (p = 0.02). This pattern was also observed for basal insulin at 16.3 (6.2) vs 23.4 (11.0) IU (p = 0.007) and bolus insulin at 22.6 (12.3) vs 35.4 (15.8) IU (p = 0.013). The reservoir change interval was longer in the ≥5 days group 6.27 (2.3) vs 3.48 (0.9) days (p < 0.001). No differences were observed according to prior infusion set type, insulin type, or MiniMed 780G configuration.

**Fig. 2:**
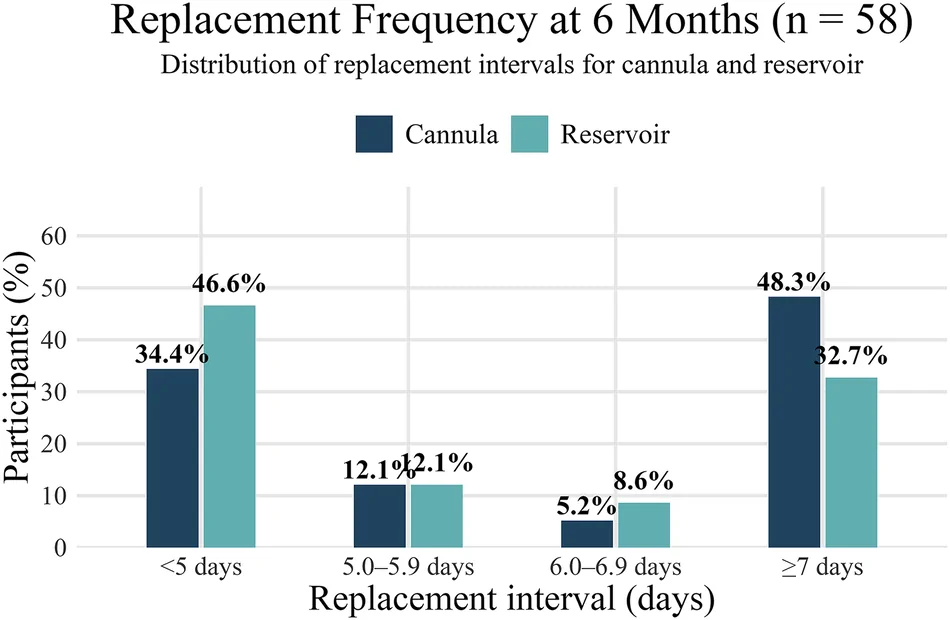
Infusion set replacement intervals at 6 months. Values represent the mean replacement interval per participant, calculated across all infusion sets and reservoir changes recorded during the final follow-up period and assessed at the 6-month visit

At the end of the study at 6 months, 48.3% of participants maintained the full 7-day wear duration. An additional 5.2% wore the set for 6–6.9 days, 12.1% for 5–5.9 days, and the remaining participants for shorter durations. A similar distribution was observed for reservoir change intervals (Fig. 2). Cannula wear duration was strongly correlated with reservoir change interval at 6 months (Pearson r = 0.712, p < 0.001).

## Discussion

To our knowledge, this is the first real-world study evaluating the Medtronic Extended infusion set in adults with T1DM using the MiniMed 780G AID system. Our findings demonstrate that switching to this device significantly increased catheter wear duration, while remaining safe and well tolerated, and being associated with high patient satisfaction without clinically meaningful deterioration in CGM-derived glycemic metrics. In addition, continuation rates remained high after 6 months of use. However, approximately half of participants did not maintain the full 7-day wear duration.

Until recently, infusion set manufacturers recommended catheter replacement before day 3. However, this practice has not been supported by robust comparative evidence demonstrating superior effectiveness or safety. Available research remains limited and is often based on retrospective studies with small samples in which infusion set performance is not the primary endpoint. It is notable that few studies have specifically evaluated safety, satisfaction, and the potential impact of different cannula-based infusion sets on glycemic control, despite the fact that infusion set-related problems are among the most frequently reported issues in the literature and are associated with a higher burden of acute complications in users of integrated systems [17]. Further studies are needed to define optimal wear duration, identify patient characteristics associated with benefit, and evaluate new materials or antimicrobial approaches that may allow longer wear without compromising safety [18].

Our study provides real-world evidence supporting the safety of the device in users of contemporary hybrid closed-loop systems. Switching to an E-iS was not associated with an increase in severe hypoglycemia, diabetic ketoacidosis, occlusion alarms, unexplained hyperglycemia, or other catheter-related complications. Likewise, longer wear did not increase local adverse reactions, including skin or device-related events, compared with the previous 3 day-infusion sets. Rates of skin marks and wounds remained similar (41.5% and 16.9% with the previous set vs 39.7% and 17.2% with the Extended set, respectively). In addition, unexplained hyperglycemia did not increase significantly during follow-up and was reported by 23.1%, 31.1%, and 25.9% of participants at baseline, 3 months, and 6 months, respectively. Occlusion alarms also remained stable and were reported by 15.4%, 18.0%, and 8.6% of participants, respectively. Nonetheless, the frequency of skin reactions remained high throughout the study and exceeded that reported in the pivotal trial of the Medtronic Extended infusion set [14].

The prevalence of skin complications in our cohort, including skin marks in 41% and skin wounds in 17%, is consistent with international reports and aligns with the trends highlighted in the FITTER Forward 2025 consensus. This report emphasizes that dermatological and mechanical alterations at the infusion site are common in insulin pump therapy and not isolated events. It describes rates of pigmentation and scarring above 45% in large cohorts, supporting the concept of subcutaneous tissue vulnerability during continuous infusion. Our observation that almost half of participants reported skin marks underscores the importance of structured site rotation protocols and individualized selection of cannula types and materials to reduce skin-related burden [10].

In addition, use of the device was associated with high satisfaction and favorable patient-reported outcomes. We observed significant improvement in cannula insertion pain and in overall satisfaction as well as in the EsDQoL satisfaction subscale, which may be related to the reduced number of insertions required with extended wear. Only 8% of participants who completed follow-up returned to the previous infusion system.

These real-world findings are consistent with results from the only published prospective multicenter non-randomized trial specifically evaluating the Medtronic Extended infusion set, conducted with an earlier-generation AID system, the MiniMed 670 G with Guardian 3 sensor. Brazg et al. reported a low rate of unexplained hyperglycemia and no severe device-related adverse events or diabetic ketoacidosis, together with greater satisfaction with the E-iS compared with standard infusion sets, findings that are in line with our real-world observations [14].

Wear duration deserves specific consideration. In prior studies, 77.8% of users were able to maintain the catheter for the full 7 days. In our cohort, 48.3% maintained the set for the full 7-day period, indicating a lower proportion achieving the theoretical maximum in routine clinical practice, which is consistent with previous reports for infusion sets of similar intended duration [19]. Importantly, comparisons between participants who wore the set for 5 days or more and those who wore it for fewer than 5 days did not show significant differences in the evaluated outcomes, including glycemic control, adverse events, and satisfaction, which remained high in both groups. The only significant difference was total daily insulin dose, which was higher among those wearing the set for fewer than 5 days. This may reflect practical considerations, as higher insulin requirements can lead patients to synchronize reservoir replacement with infusion set changes. This interpretation is supported by the strong correlation observed between cannula wear duration and reservoir change interval at 6 months (r = 0.712, p < 0.001) as shown in Fig. 2. This pattern suggests that in routine practice infusion set changes are frequently driven by reservoir replacement rather than by catheter-related issues. These findings highlight the importance of considering insulin requirements when recommending extended-wear infusion sets in routine clinical practice and may influence cost–benefit considerations in routine care.

Although continuation remained high, the E-iS was not suitable for all participants. Five participants returned to their previous infusion set because of adhesive failure with insulin leakage, persistent nodules or induration, early hyperglycemia, or inability to maintain wear for at least 5 days. These findings suggest that, in routine clinical practice, the benefit of extended-wear infusion sets may depend on individual factors such as skin tolerance, adhesive performance, local tissue response, insulin requirements, and patient preference.

Despite these considerations, use of the extended-wear system was associated with lower prescribed insulin use related to infusion set management, while infused total daily insulin doses, including basal and bolus components, remained stable. This reduction may reflect decreased insulin waste due to fewer priming and reservoir fill procedures when using longer-wear sets. Our real-world findings are consistent with the efficiency model proposed by Kwa et al., who reported that a 7-day infusion set improves device survival and treatment-related cost-effectiveness [20]. In that analysis, extending wear from 3 to 7 days reduced consumable use and decreased insulin waste by minimizing purge volume at each change. In our cohort, stable insulin doses together with reduced insulin used for set management are in line with this operational efficiency model and suggest improved resource utilization with the E-iS. This is clinically relevant because the intervention may contribute not only to higher treatment satisfaction but also to optimizing resource use in routine practice.

Similarly, improvements in satisfaction and overall metabolic control during follow up were not associated with evaluated covariates, including insulin type, prior infusion set model, or MiniMed 780G settings. This suggests that the observed changes were not driven by these factors and may instead reflect the effect of the infusion set strategy itself.

In contrast to the study by Brazg et al., our study showed a small deterioration in key CGM metrics, including TIR, TiTR, and TAR, despite a modest improvement in HbA1c. This apparent discrepancy may reflect the different physiological meaning of these metrics. HbA1c reflects longer-term average glycemic exposure, whereas CGM metrics are more sensitive to short-term fluctuations. Therefore, modest increases in postprandial or intermittent hyperglycemia may reduce TIR and increase TAR without substantially affecting HbA1c, particularly when hypoglycemia remains unchanged. These real-world findings are consistent with FITTER Forward 2025 recommendations, which caution that local inflammation and insulin degradation at the insertion site may increase when infusion sets are worn beyond 72 h [10]. However, it is important to emphasize that the observed deterioration with E-iS was minimal and unlikely to be clinically meaningful, with changes in TIR and TiTR of approximately 2%. Moreover, we did not identify a significant association between these changes and wear duration, baseline infusion set type, or other infusion set-related variables. In addition, 84.5% of participants maintained TIR above 70%, consistent with recommended targets in international clinical guidelines [21]. Importantly, no increase in severe hypoglycemia or DKA was observed during follow-up. More recently, a prospective multicenter home-use study evaluated an investigational 7-day extended-wear infusion set in adults with T1DM using a different pump and infusion set system, specifically the SteadiSet infusion set with the t:slim X2™ insulin pump with Control-IQ™ technology [22]. Although these findings support the broader feasibility of extended-wear infusion set strategies, direct comparison with our real-world Medtronic MiniMed 780G cohort should be interpreted with caution because a different infusion set, AID technology, and study setting were used.

This study has several strengths. To our knowledge, it is the first real-world evaluation of the Extended infusion set in users of the contemporary AID system: Minimed 780G. Participants had substantial prior experience with insulin pump therapy and hybrid closed loop systems. All participants received standardized therapeutic education and structured follow up combining in person and telephone contacts. Prospective data collection using validated questionnaires enabled a multidimensional assessment. The study also provides information on a topic that is often underreported in routine care, namely infusion set tolerability and adverse events, and it evaluates persistence with the E-iS over the medium term, which is particularly relevant for real-world implementation.

Limitations should also be acknowledged. This was a single-center prospective non-randomized study conducted in a tertiary care hospital in participants using a single hybrid closed loop system and with high adherence and high time in automatic mode. These factors may limit generalizability. Although a sample size calculation was performed, the overall sample remained relatively modest. In addition, the absence of a parallel control group limits causal inference

In conclusion, our study supports extended wear infusion sets as a safe and effective strategy that prolongs cannula wear and increases reservoir change intervals without increasing severe acute events, including severe hypoglycemia or diabetic ketoacidosis requiring hospitalization, while maintaining overall glycemic control without clinically meaningful deterioration. The Extended set was associated with greater satisfaction and improved tolerability, including lower insertion pain, with stable quality of life assessed by validated questionnaires and high medium-term persistence. These findings suggest that extended wear infusion sets represent a practical option to reduce the burden of infusion set changes in AID users, although individual tolerance and the ability to maintain prolonged wear should be considered when selecting candidates for this strategy.

## Abbreviations

AID: Automated insulin delivery
BMI: Body mass index
CGM: Continuous glucose monitoring
CSII: Continuous subcutaneous insulin infusion
CV: Coefficient of variation
DKA: Diabetic ketoacidosis
E-iS: Extended infusion set
EsDQoL: Spanish version of the Diabetes Quality of Life questionnaire
FDA: Food and Drug Administration
GMI: Glucose management indicator
HbA1c: Glycated hemoglobin
NGSP: National Glycohemoglobin Standardization Program
T1DM: Type 1 diabetes mellitus
TAR: Time above range
TBR: Time below range
TiTR: Time in tight range
TIR: Time in range
TDD: Total daily insulin dose
VAS: Visual analog scale

## References

[1] American Diabetes Association Professional Practice Committee, 2. Diagnosis and Classification of Diabetes: Standards of Care in Diabetes-2026. Diabetes Care **49**, S27–S49 (2026). 10.2337/dc26-S002 41358893 10.2337/dc26-S002 PMC12690183

[2] D. Tomic, J.E. Shaw, D.J. Magliano, The burden and risks of emerging complications of diabetes mellitus. Nat Rev Endocrinol **18**, 525–539 (2022). 10.1038/s41574-022-00690-7 35668219 10.1038/s41574-022-00690-7 PMC9169030

[3] American Diabetes Association Professional Practice Committee, 7. Diabetes Technology: Standards of Care in Diabetes-2026. Diabetes Care **49**, S150–S165 (2026). 10.2337/dc26-S007 41358889 10.2337/dc26-S007 PMC12690173

[4] Rivero-Santana A, Ramos-García V, Toledo-Chávarri A, García-Pérez L, Valcárcel-Nazco C, González-Pacheco H, et al. Efectividad, seguridad y coste efectividad de los sistemas de monitorización continua de glucosa en tiempo real, sistemas de asa abierta y sistemas híbridos de asa cerrada (páncreas artificial) para pacientes con diabetes [Internet]. Madrid: Ministerio de Sanidad. Servicio de Evaluación del Servicio Canario de la Salud. 2023 [citado 18 de noviembre de 2024]. 468 p. Informes de Evaluación de Tecnologías Sanitarias.

[5] S. Di Molfetta, L. Di Gioia, I. Caruso, A. Cignarelli, S.C. Green, P. Natale et al. Efficacy and safety of different hybrid closed loop systems for automated insulin delivery in people with Type 1 diabetes: A systematic review and network meta-analysis. Diabetes Metab Res Rev **40**(6), e3842 (2024). 10.1002/dmrr.3842 39298688 10.1002/dmrr.3842

[6] B.A. Buckingham, G.P. Forlenza, J.E. Pinsker et al. Automated insulin delivery systems: from early research to routine care of type 1 diabetes. Curr Diab Rep **22**(7), 345–352 (2022)

[7] M.S. Hughes, B.A. Buckingham, G.P. Forlenza, S.K. Garg, F.J. Doyle 3rd, D.M. Maahs et al. Frequency and detection of insulin infusion site failure in insulin pump users including automated insulin delivery. J Diabetes Sci Technol **17**(4), XXXX–XXXX (2023)

[8] Medtronic. Un equipo para cada persona: Equipos de infusión Medtronic [Internet]. Medtronic Diabetes; 2022 [citado 2025 May 12]. Disponible en: https://www.medtronicdiabetes.com/sites/default/files/library/download-library/user-guides/un-equipo-para-cada-persona-equipos-de-infusi%C3%B3n-medtronic.pdf

[9] P. Choudhary, R. Kolassa, W. Keuthage, J. Kroeger, C. Thivolet, M. Evans et al. ADAPT study Group. Advanced hybrid closed loop therapy versus conventional treatment in adults with type 1 diabetes (ADAPT): a randomised controlled study. Lancet Diabetes Endocrinol **10**(10), 720–731 (2022). 10.1016/S2213-8587(22)00212-1.Erratum. in: Lancet Diabetes Endocrinol. 2023 Jul;11(7):e9. doi: 10.1016/S2213-8587(23)00146-8. PMID: 36058207 36058207 10.1016/S2213-8587(22)00212-1

[10] D.C. Klonoff, L. Berard, D.R. Franco, S. Gentile, O.V. Gomez, Z. Hussein et al. FITTER Forward Expert Recommendations. Advance insulin injection technique and education with FITTER Forward expert recommendations. Mayo Clin Proc **100**(4), 682–699 (2025). 10.1016/j.mayocp.2025.01.004 40180487 10.1016/j.mayocp.2025.01.004

[11] M. Cescon, D.J. DeSalvo, T.T. Ly, D.M. Maahs, L.H. Messer, B.A. Buckingham et al. Early detection of infusion set failure during insulin pump therapy in type 1 diabetes. J Diabetes Sci Technol **10**(6), 1268–1276 (2016). 10.1177/1932296816663962 27621142 10.1177/1932296816663962 PMC5094340

[12] A.L.D. Neves, L.E.G. Martins, M.A.L. Gabbay, P. Pascali, T. de Oliveira, A. Martinazzo et al. Insulin infusion sets associated with adverse events: strategies for improved diabetes education. Front Med (Lausanne) **10**, 1275394 (2023). 10.3389/fmed.2023.1275394 38093983 10.3389/fmed.2023.1275394 PMC10716844

[13] P. Dekker, H.J. Aanstoot, T. Sas, M. de Vries, E. Birnie, D. Mul, G. Nefs, Prevalence of and reasons for discontinuation of continuous subcutaneous insulin infusion in people with Type 1 diabetes: a systematic review. Diabetes Technol Ther **25**(8), 559–570 (2023). 10.1089/dia.2023.0038 37053533 10.1089/dia.2023.0038

[14] R. Brazg, S.K. Garg, A. Bhargava, J.R. Thrasher, K. Latif, B.W. Bode et al. Evaluation of extended infusion set performance in adults with Type 1 diabetes: infusion set survival rate and glycemic outcomes from a pivotal trial. Diabetes Technol Ther **24**(8), 535–543 (2022). 10.1089/dia.2021.0540 35263188 10.1089/dia.2021.0540 PMC9353978

[15] M. Millan, Cuestionario de calidad de vida específico para la diabetes mellitus (EsDQOL). Aten Primaria **29**, 517–521 (2002). 10.1016/s0212-6567(02)70623-9. Spanish 12031227 10.1016/S0212-6567(02)70623-9 PMC7679554

[16] M. -Jansa, C. Quirós, M. Giménez, M. Vidal, M. Galindo, I. Conget, Análisis psicométrico de las versiones en lengua castellana y catalana de un cuestionario de percepción de la hipoglucemia. Med Clin (Barc) **144**(10), 440–444 (2015). 10.1016/j.medcli.2015.05.013. Spanish 24529399 10.1016/j.medcli.2013.11.036

[17] L. Heinemann, L. Krinelke, Insulin infusion set: the Achilles heel of continuous subcutaneous insulin infusion. J Diabetes Sci Technol **6**, 954–964 (2012). 10.1177/193229681200600429 22920824 10.1177/193229681200600429 PMC3440169

[18] G. Zhang, O. Cohen, S. Chattaraj, Development of the extended infusion set and its mechanism of action. J Diabetes Sci Technol **18**(2), 454–459 (2024). 10.1177/19322968221112120 35876264 10.1177/19322968221112120 PMC10973872

[19] D. Waldenmaier, E. Zschornack, A. Buhr, S. Pleus, C. Haug, G. Freckmann, A prospective study of insulin infusion set use for up to 7 days: early replacement reasons and impact on glycemic control. Diabetes Technol Ther **22**, 734–741 (2020). 10.1089/dia.2019.0445 32167382 10.1089/dia.2019.0445

[20] T. Kwa, G. Zhang, K. Shepard, K. Wherry, S. Chattaraj, The improved survival rate and cost-effectiveness of a 7-day continuous subcutaneous insulin infusion set. J Med Econ **24**(1), 837–845 (2021). 10.1080/13696998.2021.1945784 34154504 10.1080/13696998.2021.1945784

[21] T. Battelino, T. Danne, R.M. Bergenstal, S.A. Amiel, R. Beck, T. Biester et al. Clinical targets for continuous glucose monitoring data interpretation: recommendations from the international consensus on time in range. Diabetes Care **42**(8), 1593–1603 (2019). 10.2337/dci19-0028 31177185 10.2337/dci19-0028 PMC6973648

[22] R.A. Lal, J.W. Lum, Z.W. Reed, D. Kruger, J.C. Reed 3rd, R.S. Weinstock et al. A multicenter study of an investigational extended wear insulin infusion set in adults with Type 1 diabetes. Diabetes Technol Ther **28**(1), 1–9 (2026). 10.1177/15209156251364548 40780817 10.1177/15209156251364548 PMC12912291

